# Hydrogen production and hydrogen utilization in the rumen: key to mitigating enteric methane production

**DOI:** 10.5713/ab.23.0294

**Published:** 2023-12-29

**Authors:** Roderick I. Mackie, Hyewon Kim, Na Kyung Kim, Isaac Cann

**Affiliations:** 1 Department of Animal Sciences, University of Illinois, Urbana, IL 61801, USA; 2 Carle R. Woese Institute for Genomic Biology, University of Illinois, Urbana, IL 61801, USA; 3 Department of Microbiology, University of Illinois, Urbana, IL 61801, USA

**Keywords:** Enteric Methane Mitigation, Formate, Hydrogen, Interspecies Hydrogen Transfer, Methane, Rumen

## Abstract

Molecular hydrogen (H_2_) and formate (HCOO^−^) are metabolic end products of many primary fermenters in the rumen ecosystem. Both play a vital role in fermentation where they are electron sinks for individual microbes in an anaerobic environment that lacks external electron acceptors. If H_2_ and/or formate accumulate within the rumen, the ability of primary fermenters to regenerate electron carriers may be inhibited and microbial metabolism and growth disrupted. Consequently, H_2_- and/or formate-consuming microbes such as methanogens and possibly homoacetogens play a key role in maintaining the metabolic efficiency of primary fermenters. There is increasing interest in identifying approaches to manipulate the rumen ecosystem for the benefit of the host and the environment. As H_2_ and formate are important mediators of interspecies interactions, an understanding of their production and utilization could be a significant starting point for the development of successful interventions aimed at redirecting electron flow and reducing methane emissions. We conclude by discussing in brief ruminant methane mitigation approaches as a model to help understand the fate of H_2_ and formate in the rumen ecosystem.

## BACKGROUND

Animal agriculture has been identified as one of the major sources of greenhouse gases (GHGs) accounting for approximately 40% of the total agriculture related emissions. Animal production and manure management comprise 26.8% and 31.0% respectively of the 7.1 Gt of CO_2_ equivalents that the livestock sector is estimated to produce annually [1,2]. The two main GHGs emitted directly from animal agriculture include methane (CH_4_) and nitrous oxide (N_2_O) which have 28 and 298 times the global warming potential of CO_2_ respectively. Livestock CH_4_ and N_2_O emissions have been estimated to contribute 40% and 48% of livestock sector emissions where ruminants account for 80% of the total livestock sector’s emissions [1]. Although all livestock contribute to total GHG emissions, in this review we focus on enteric CH_4_ emissions from ruminant animals that result from anaerobic fermentation in the rumen that harbor methanogenic archaea that effectively use the hydrogen produced during saccharolytic fermentation to reduce CO_2_ and produce methane gas that is eructated out of the rumen and contributes to global warming.

Agriculture is the largest single source of global anthropogenic methane emissions with ruminants the dominant contributor. Globally, methane emissions account for 40% to 45% of greenhouse gas emissions from ruminant livestock with over 90% of these emissions arising from enteric fermentation [2,3]. About 80% of agricultural CH_4_ arises from livestock systems, of which almost 90% comes from enteric fermentation by ruminants such as cattle and sheep, and about 10% from animal manure [4]. The remaining 20% of agricultural emissions arises primarily from rice paddies. Livestock CH_4_ emissions are projected to grow another 30% by 2050 due to a growing human population and increasing demand for animal protein as incomes rise although with significant variations in demand and economic trends by region and country. The relative contributions of CH_4_ and CO_2_ over the short and long term are still being debated but given that CO_2_ persists in the atmosphere for 300 to 1,000 years, and therefore accumulates over time. In contrast, CH_4_ has an atmospheric lifetime of approximately 12.5 years and therefore emissions do not accumulate over centuries. However, even a moderate reduction in global CH_4_ emissions at a rate of about 0.3% per year would stabilize warming from CH_4_ at current levels [3]. However, it is obvious that net CO_2_ emissions need to drop to zero for temperature to stabilize and additional warming will occur until that condition is reached. It has been argued that expressing CH_4_ emissions as CO_2_ equivalents i.e. 28 times the warming potential of CO_2_, is both misleading and dangerous as it shifts attention away from the need to reduce global CO_2_ emissions with a half-life that is many times longer than CH_4_. In addition, the biological origin of livestock CH_4_ emissions that are part of a natural renewable cycle starting with photosynthesis and plant growth and therefore fundamentally different from burning fossil fuels for example. Nevertheless, if the contribution of global food systems to CH_4_ emissions over a 20-year period is considered instead of the normal 100-year period the contribution of CH_4_ to food systems, GHG emissions double and it is imperative to reduce that both CH_4_ and N_2_O emissions from livestock are reduced significantly to achieve the goal of the Paris Agreement to limit global warming to 1.5°C above pre-industrial levels [3].

## CARBON AND ELECTRON FLOW IN ANAEROBIC DIGESTORS AND GUT SYSTEMS

Biological methanogenesis is an important part of the global carbon cycle and occurs in a range of different habitats that include marshes, freshwater and marine sediments, rice paddies, geothermal habitats, anerobic bioreactors and the topic of this review, the rumen ecosystem. During the process anaerobic degradation of complex organic matter results in generation of gaseous products (CH_4_ and CO_2_ also known as biogas) with a relatively small growth yield of bacteria, but with 90% or more of the energy retained in CH_4_ [5]. This process occurs in environments where the main electron acceptors, such as CO_2_, that are involved in degradation are also generated from the organic substrates degraded. This process does not occur in environments where electron acceptors such as oxygen, nitrate or sulfate are readily available and outcompete H_2_-consuming methanogens for H_2_.

**Figure 1:**
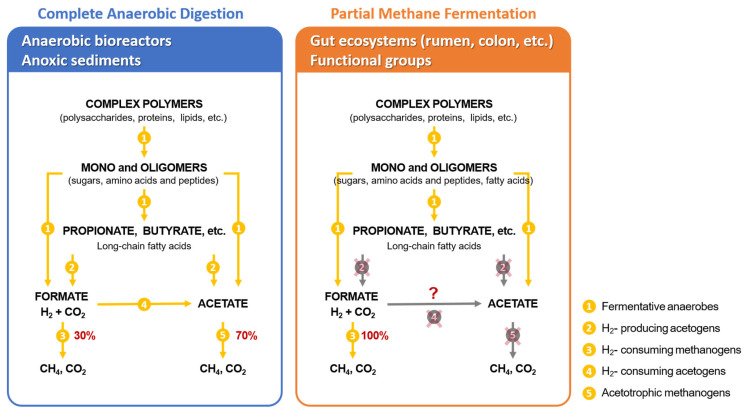
Three stage scheme for the complete degradation of organic matter (in the left panel) showing the general sequence and major metabolic groups of bacteria. The panel on the right shows partial anaerobic degradation found in the rumen and other mammalian gut systems where kinetic constraints of rapid turnover prevent the breakdown of volatile fatty acids which are instead absorbed by the host to supply metabolizable energy and form the basis for the unique symbiosis between the rumen, its microbes and the herbivorous host [6,21].

There is an important distinction in the flow of carbon to CH_4_ between systems that have long residence times like anaerobic bioreactors (>14 to 20 days) and sediments (years and decades) compared to intestinal systems where turnover occurs once or twice per day. Thus, in the gastrointestinal tracts of ruminants and in the large intestine of other animals, including the human colon, only a partial methane fermentation occurs. This means that the intermediate volatile fatty acids accumulate and are absorbed from the intestinal tract and serve as energy substrates and metabolites to the host animal in a symbiotic interaction. In order to simplify the discussion of the chemistry, microbiology and kinetics of fermentation it is instructive and informative to discuss stages of fermentation (Figure 1) [5,6]. Generally, the first stage is considered to involve the hydrolytic and fermentative bacteria that breakdown polymers such as cellulose, hemicellulose and pectin that occur in plant cell walls and ferment them to organic acids, alcohols, H_2_ and CO_2_. Proteins and lipids are also fermented to similar products but also include NH_3_ and H_2_S. The second stage bacteria named the H_2_-producing acetogenic bacteria that obtain energy for growth by producing acetate and H_2_ from the organic acids and alcohol produced in the first stage. The third stage involves the methanogenic archaea that utilize the products of the first two stages, mainly H_2_, CO_2_ and acetate, to generate the final products methane and CO_2_. The important difference in partial methane fermentation as occurs in the rumen, large intestine of animals and the human colon is that only the bacteria and archaea involved in the first and last stages of the 3-stage scheme are present and functional. As a consequence of the differences in the pattern of carbon flow in these two systems, about 60% to 70% of the CH_4_ generated in bioreactors is derived from the methyl group of acetate and aceticlastic methanogenesis dominates whereas in rapidly turning over gut systems, such as the rumen, most of the methane (>95%) is derived from hydrogen driven reduction of CO_2_ i.e. hydrogenotrophic methanogenesis.

The microbiological explanation for this is that, because of the relatively short retention time of digesta in the intestinal tract, the H_2_-producing acetogenic bacteria which catabolize fatty acids as well as the acetate catabolizing aceticlastic methanogens are unable to grow fast enough to keep up with dilution rate and are not maintained in the rumen. Faster dilution rates are driven by consumption of fresh organic matter and the passage of digesta through the rumen and the reticulo-omasal orifice to the abomasum and small intestine. Importantly, acetate and other short chain fatty acids such as propionate and butyrate accumulate in the respective fermentation compartment and are subsequently absorbed and serve as major energy sources to the host animal in a unique symbiotic relationship. While the stages of fermentation can be schematically separated this is not the case for their metabolism where the metabolism of each group is highly dependent on interactions between the others.

**Figure 2:**
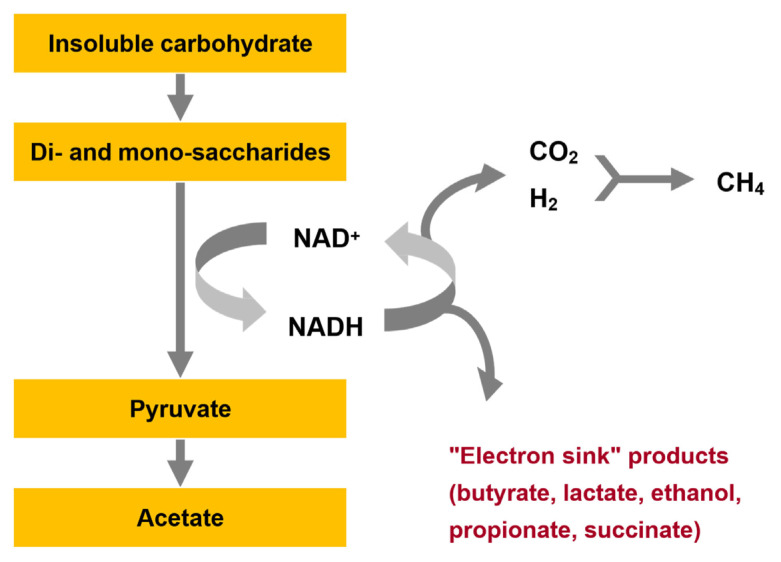
Biochemical degradation pathway for polysaccharides via the Embden-Meyerhoff glycolytic pathway showing the critical metabolic and regulatory role that reoxidation of nicotinamide adenine dinucleotide (NADH) generated during glycolysis by methanogenesis and the formation of electron sink products. Regeneration of NAD allows the glycolytic sequence to proceed efficiently.

**Figure 3:**
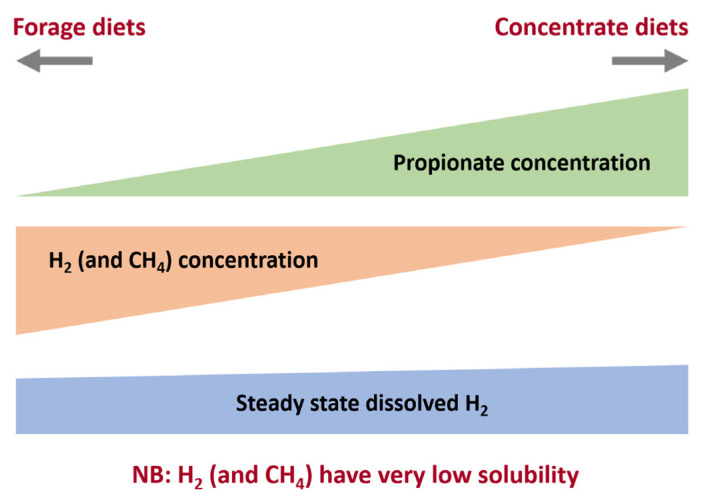
Relationship between H_2_, CH_4_, and propionate concentrations in the rumen. High forage diets result on a fermentation balance with high acetate and low propionate and produce more CH_4_ per mole hexose fermented than high concentrate diets with higher propionate:acetate ratios. It is worth noting that H_2_ solubility is very low and thus dissolved H_2_ concentrations do not shown the same change as volatile fatty acids ratios.

There are two levels in the anaerobic fermentation scheme (Figure 1) where hydrogen plays an important regulatory role [5,6]. The first is during the first stage of fermentation carried out by the fermentative bacteria. In this scheme sugars are fermented mainly by the Embden-Meyerhof-Parnas pathway to pyruvate. Generating electrons designated as 2H although this is in the form of nicotinamide adenine dinucleotide (NADH). The pyruvate is then further catabolized to acetate, CO_2_ and H_2_, or to electron sink products propionate, butyrate, lactate and even ethanol occasionally (Figures 2 and 3). Succinate is likely an important extracellular intermediate as it is an end-product of important fermentative bacteria and is decarboxylated to propionate by other species. Metabolic interactions among fermentative bacteria and between H_2_-producing bacteria and H_2_-utilizing methanogens show that the concentration of H_2_ plays a central role in regulating the proportions of the various end-products produced by the fermentative bacteria. The thermodynamic explanation for this is based on NAD-linked H_2_ formation as shown in the equation below:


$$\text{NADH}+{\text{H}}^{+}\to \text{NAD}+{\text{H}}_{2}\;\;\;\Delta {\text{G}}_{\text{o}}^{\prime }=+4.3\;\text{kcal}/\text{reaction}$$

The oxidation of NADH with H _2_ production is essential for degradation of organic matter to proceed but the equilibrium of reaction is in the direction of NADH formation unless the partial pressure of H_2_ is maintained at a very low level. This is achieved by efficient metabolism of H_2_ by methanogenic archaea. When H_2_ concentrations increase when the glycolytic flux is increased by feeding high proportions of readily fermentable carbohydrate in ruminant diets or when anaerobic bioreactors are stressed by increasing the organic loading rate or shortening the retention time (increasing dilution rate) in anaerobic bioreactors there is a shift towards increased metabolism of pyruvate to reduced products especially propionate rather than acetate, CO_2_ and H_2_. Other more reduced end-products such as butyrate, and valerate as well as lactate and even ethanol. Thus in an efficiently operating methanogenic system where the partial pressure of H_2_ is maintained at a very low level most carbohydrate is fermented to acetate and less to more reduced end-products.

Another way of looking at metabolic interactions and proportions of volatile fatty acids (VFAs) produced is provided by calculating the stoichiometric amount of methane produced per mole of hexose fermented in mixed acid fermentation such as the rumen [7]. In this theoretical approach, a set of equations was developed based on knowledge of fermentations of individual rumen bacterial species, information about interactions between species, as well as general knowledge of bacterial fermentations. Calculations based on these equations were used to calculate the moles of CH_4_ produced per mole of hexose fermented for different proportions of VFAs in the rumen shown below (Table 1) and demonstrate the quantitative importance of a shift in fermentation towards more reduced end-products such as propionate and butyrate (Figure 3).

Thus, changes in populations responsible for the fermen tations can influence the proportions of VFAs produced by carbohydrate fermentation and importantly alter the amount of CH_4_ produced per mole of hexose fermented and the formation of CH_4_ is closely associated with the proportions of VFA formed. Importantly, when determining a H fermentation balance it is important to account for 2H consumed in changes to the VFAs production profile. In the rumen fermentation methanogenesis was estimated to be the main [H] sink with propionate second in importance [8].

The second level where H _2_ plays an important regulatory role is the intermediate stage carried out by H_2_-producing acetogenic bacteria. In this step of the anaerobic fermentation scheme (Figure 1), propionate and longer chained saturated fatty acids are anaerobically oxidized to acetate, CO_2_ and H_2_. In most cases the electron sink is H_2_ but it is also possible that the electrons generated could be via formate production by reactions involving reduction of CO_2_. The formate could then be utilized directly by methanogens, or after being degraded to CO_2_ and H_2_. In the rumen fermentation, it was estimated that 18% of rumen CH_4_ was produced from formate as [H] donor [9]. The metabolic interactions involving obligate interspecies hydrogen transfer (IHT) also called obligate syntrophism, in which the H_2_-producer cannot grow carrying out a given reaction unless a H_2_-consunmer maintains a low enough H_2_-partial pressure to allow the reaction to be thermodynamically possible. The effects of H_2_-partial pressure on the ΔG’_o_ of several H_2_-producing and H_2_-consuming reactions involved in obligate interspecies H_2_ transfer can be explained thermodynamically. Thus for H_2_-consuming methanogenic archaea and sulfate reducers the ΔG’_o_ for the respective reactions increases (becomes less favorable) as H_2_ partial pressure decreases. In contrast, for the H_2_-producing reactions, the ΔG’_o_ values decrease (become more favorable) as the H_2_-partial pressure deceases becoming increasingly favorable. Since both the H_2_-producing and H_2_-consuming organisms must conserve energy the H_2_ level must be poised at a partial pressure defined by the boundary conditions for the coupled reaction i.e. in a window of 10^−2^ and 10^−5^ atm of H_2_-partial pressure. These constraints therefore control the oxidation of ethanol, butyrate and propionate in the complete anaerobic degradation of organic matter (Figure 1). There are also examples of facultative interspecies H_2_ transfer in which the H_2_-producer benefits from the interaction but does not require it. Pure cultures of some anaerobic bacteria can produce H_2_ from pyruvate oxidation to acetyl-CoA and CO_2_ because ferredoxin has a redox potential of −400 mV close to that of H_2_ (−414 mV at pH = 7). In contrast NADH has a potential of −320 mV and is not a strong enough reductant to produce H_2_ under standard condition (1 atm). However, if the H_2_ partial pressure is kept at <10^−3^ (ca. 320 mV) then H_2_ production from NADH is favorable. This allows hydrogenase expressing anaerobic bacteria to dispose of electrons from NADH to H_2_ rather than having to dispose of them on pyruvate and producing electron sink products such as lactate or ethanol. This means that this interaction between anaerobes allows all of the pyruvate to be oxidized to acetyl-CoA which can then be conserved as ATP via acetyl-phosphate. Furthermore, one should consider typical fermentation products seen in pure culture fermentations as the products of unbalanced metabolism in the absence of H_2_ consumers.

Methanogenesis is a terminal process in anaerobic biomass degradation, common in habitats where terminal electron acceptors, such as oxygen, nitrate, iron(III), and sulfate, are missing or rapidly depleted [10,11]. The major substrates for methanogenesis are CO_2_, acetate, and methylated compounds. Hence, three main methanogenesis pathways are distinguished: i) CO_2_-reducing (hydrogenotrophic); ii) aceticlastic; and iii) methylotrophic methanogenesis. In methylotrophic methanogenesis, the methyl-groups of methylated compounds such as methanol, methylamines, and methylated sulfides are transferred to substrate-specific corrinoid proteins and further to CoM to finally be reduced to CH_4_.

### H_2_-dependent methylotrophs

Until recently, little attention was paid to methylotrophic methanogenesis as it was presumed to be less common among methanogens than hydrogenotrophic methanogenesis and globally less important than aceticlastic methanogenesis. However, this concept has changed since the discovery of the *Methanomassiliicoccales* [12], previously called rumen cluster C (RCC) and *Methanoplasmatales* groups before the new taxonomic name was accepted. *Methanomassiliicoccales* constitute a major proportion of the methanogens in the rumen [13–15]. In metatranscriptomic studies of the bovine rumen microbiome, it was shown that they were highly active and abundant [16], especially after feeding [17]. *Methanomassiliicoccales* were also identified as transcriptionally active in the ovine rumen [18,19]. Due to their capability of utilizing methylamines as methanogenesis substrates, *Methanomassiliicoccales* occupy a previously unnoticed ecological niche in the rumen [16]. Furthermore, ammonium is produced as an end-product of methylamine reduction and likely serves as a nitrogen source for other rumen microorganisms [16]. In contrast, they compete with other methanogens for the electron donor H_2_ and they compete for methanol with *Methanosphaera* species. Based on transcriptomic data, *Methanomassiliicoccales* may avoid competition with *Methanosphaera* spp. by utilizing methylamines, when available. In general, hydrogenotrophic methanogens, mainly *Methanobrevibacter* sp. (*Methanobacteriales*), are considered as dominant CH_4_ producers in ruminants [20,21]. In addition, CO_2_ is a much more abundant methanogenic substrate than methylamines and methanol. Thus, it is somewhat surprising that *Methanomassiliicoccales* are so abundant and active. Taking this into account, rumen *Methanomassiliicoccales* appear as potent targets for CH_4_ mitigation strategies [16,17].

## INTERSPECIES HYDROGEN TRANSFER

The mutually beneficial interdependence of hydrogen-producing and hydrogen-utilizing bacteria was discovered by M. P. Bryant, M. J. Wolin, and R. S. Wolfe at the University of Illinois in 1967 [22]. Based on thermodynamic principles, IHT is a central process in anaerobic environments linking transfer of reducing power from fermentation of organic molecules to inorganic electron acceptors via hydrogen. Interspecies hydrogen transfer is the most significant example of unidirectional substrate supply enabling the syntrophic metabolic association between interacting microbial species and plays a significant role in the global methane cycle. Hydrogenases are essential to IHT. They catalyze the reversible reduction of protons coupled to the oxidation of H_2_ [23,24].


$$2{\text{e}}^{-}+2{\text{H}}^{+}↔{\text{H}}_{2}$$

Hydrogenases contain one of three metal centers ([NiFe], [FeFe], or [Fe]), which bind hydrogen and occur across all domains of life [23,25]. The [NiFe]-hydrogenases are the most diverse and widespread of these groups [23]. The [FeFe]-hydrogenases are much less well understood [23]. Hydrogenase reactions are coupled to other redox reactions such as the oxidation or reduction of NADH/NAD^+^, Fd_red_/Fd_ox_, and/or butyryl-CoA/crotonyl-CoA; the direction of the hydrogenase (hydrogenogenic or hydrogenotrophic) is dependent on the environment [26,27].

A good mechanistic example of intracellular electron flow and balance is provided by the study of *Ruminococcus albus* 7, a hydrogen-producing, fermentative bacterium with two known hydrogen-producing hydrogenase complexes, HydABC and HydA2, as well as a putative hydrogen-sensing protein, HydS [27]. HydABC is the only chromosomal hydrogenase, while HydA2 and HydS form a transcriptional unit on its plasmid pRumal01. The electron-bifurcating ferredoxin- and NAD-dependent [FeFe]-hydrogenase, HydABC, couples proton reduction using NADH to proton reduction using reduced ferredoxin (Fd_red_), producing molecular hydrogen: 3 H^+^ + NADH + Fd_red_ → 2 H_2_ + NAD^+^ + Fd_ox_. HydA2, a ferredoxin-dependent [FeFe]-hydrogenase, reduces protons to molecular hydrogen using only reduced ferredoxin: 2 H^+^ + Fd_red_ → H_2_ + Fd_ox_. HydS contains a PAS domain, which often are present on sensory proteins. In addition, HydS contains a putative redox-sensing [4Fe:4S] cluster.

For our co-culture studies, we hypothesized HydS tran scriptionally regulates HydA2 in a manner dependent on the presence of a hydrogen-utilizing syntroph [28]. To test this hypothesis, *R. albus* 7 and a hydrogen-utilizing bacterium, *Wolinella succinogenes* DSM 1740, were grown in pure culture and in co-culture. *W. succinogenes* uses hydrogen as an electron acceptor for fumarate respiration [28]. Cell growth was monitored by optical density (OD_600_) and quantitative polymerase chain reaction. Metabolites were measured to observe changes caused by the interaction of the two bacteria. Lastly, RNA was extracted at mid-log phase for sequencing to compare whole genome transcriptomic profiles. Hydrogen accumulated in the *R. albus* pure culture, but not in the co-culture. Production of acetate increased and ethanol decreased when *R. albus* was grown in co-culture with *W. succinogenes*. Transcript abundance of HydA2 was 90-fold lower in co-culture, relative to pure culture. The electron-bifurcating hydrogenase, HydABC, had a small change in transcript abundance in co-culture relative to pure culture (1.2- to 1.3-fold increase). This suggests HydS might be sensing hydrogen levels and regulating the transcription of HydA2. These results also suggest the electron-bifurcating hydrogenase (HydABC) functions in central metabolism regardless of external hydrogen concentration. In addition, many genes in central carbon metabolism, *de novo* thiamin biosynthesis, and methionine transport were significantly increased.

*W. succinogenes* reduced all the fumarate to succinate in both the pure culture and the co-culture with *R. albus*. Two of the three subunits of the [NiFe]-hydrogenase in *W. succinogenes* had an increase in transcript abundance of 2.7-fold to 2.9-fold. The transcripts for fumarate reductase had a small increase in abundance in co-culture (1.2-fold). *W. succinogenes* had an increased growth rate in co-culture. Other respiratory genes in *W. succinogenes* had increased transcriptional abundance, including formate dehydrogenase and genes involved in nitrate reduction. Transcripts for fumarate respiration were much higher than for nitrate respiration. This is the first study to show at the genome and metabolite levels that *R. albus* and *W. succinogenes* benefit from symbiotic IHT, although formate transfer may have occurred in co-culture as well.

## RUMEN FERMENTATION

The ruminant rumen-reticulum is a large, pre-gastric fermentation organ in which mutualistic microbial fermentation takes place prior to gastric digestion [29]. The rumen is inhabited by a diverse microbial community comprised of anaerobic bacteria, methanogenic archaea, ciliate protozoa, anaerobic phycomycete fungi and bacteriophage and is known to be highly adaptable metabolically to deal with changes in diet. The rumen is characterized by the presence of a large eukaryal population of ciliate protozoa that accounts for as much as 50% of the microbial biomass. The microbes of the intestinal tract especially the rumen play vital roles in the nutritional, physiological, immunological, protective, and developmental functions of their respective hosts, however the forces that control and shape the composition and activities of these microbial communities remain poorly understood.

A major difference between the rumen and the colon is that in the rumen the microbes initiate feed degradation i.e. foregut fermentation, while in the colon the host digestive processes act on the feed first i.e. hindgut fermentation [30]. The diet of farmed ruminants is largely composed of fiber (cellulose, hemicelluloses, and pectin) and starch in varying proportions depending on the production system with a relatively constant daily intake. The human diet is highly variable and the fermentation substrates which reach the colon include undigested dietary polysaccharides such as fiber, resistant starch, and oligosaccharides that escape digestion in the upper tract. Host-secreted mucin glycans are also an important substrate for human gut microbes [31]. Rumen microbes are not thought to use host glycans, but the presence of host glycan-degrading enzymes in some rumen *Prevotella* spp. [29] suggests they may be able to use salivary glycoproteins.

Acetic, propionic, and butyric acids are the major VFA products of fermentation in both the rumen and human colon. It is well established that rumen VFAs are absorbed and contribute about 70% of the animals metabolizable energy requirement [32]. VFAs are also absorbed from the human large intestine and contribute to energy requirements of the host albeit at a lower level (~10%, [32]) An important difference lies in the production of gaseous products. Intestinal gases of humans [33] have a lower percentage of CO_2_ and CH_4_ and a greater percentage of H_2_ than gases found in the rumen. CH_4_ emission is universal in rumen fermentation, whereas the proportion of humans identified as CH_4_ emitters varies [34,35], with 20% of Western populations identified as high emitters [36]. Moreover, H_2_ is rarely a final product of rumen fermentation but is always a product of large intestinal fermentation in humans and significant amounts of residual H_2_ that is not used by microbes are excreted via expiration or flatus.

## HYDROGEN AND FORMATE METABOLISM IN THE RUMEN

H_2_ is primarily produced during microbial fermentation by hydrogenases. These enzymes catalyze the reoxidation of cofactors reduced during carbohydrate fermentation [8] and dispose of the derived electrons by reducing protons to produce H_2_. In the rumen most of the H_2_ produced is used by methanogenic archaea to reduce CO_2_ (hydrogenotrophic methanogenesis) or methyl compounds (methylotrophic methanogenesis) to CH_4_, via a process known as IHT [7]. H_2_ is maintained at sufficiently low concentrations through methanogenesis for fermentation to remain thermodynamically favorable [8,37].

A range of rumen microbes belonging to several different phyla have been shown to produce H_2_, with 65% of cultured rumen bacterial and archaeal genomes [10] encoding enzymes that catalyse H_2_ production or consumption [28]. Metagenome assembled genomes (MAGs) from different gastrointestinal tract regions of seven ruminant species [38] generated similar results. A total of 6,152 [NiFe]-, [FeFe]-, and Fe-hydrogenase-containing MAGs were detected, 3,003 of which encoded enzymes for fermentative H_2_ production (72.7% from the Firmicutes), while 95 MAGs encoded H_2_-uptake hydrogenases and the methyl-CoM reductases related to hydrogenotrophic methanogenesis (mainly from *Methanobrevibacter*).

Flavin-based electron bifurcation is an electron pair-splitting mechanism that enables the coupling of energy-producing redox reactions with energy-consuming electron transfer reactions [21] and is likely to be particularly important for fermentation in the anaerobic gut environment. Metatranscriptomic analysis using data from sheep that differed in their methane yield [22] showed that electron-bifurcating [FeFe]-hydrogenases were key mediators of ruminal H_2_ production [16]. Hydrogenases from carbohydrate-fermenting Clostridia (*Ruminococcus*, Christensenellaceae R-7 group) accounted for half of all hydrogenase transcripts, suggesting that these organisms generate much of the H_2_ used by the hydrogenotrophic *Methanobrevibacter* species. Co-culturing experiments showed that the hydrogenogenic cellulose fermenter *Ruminococcus albus* expressed its electron-bifurcating hydrogenase and suppressed its ferredoxin-only hydrogenase when grown with the hydrogenotrophic fumarate reducer *Wolinella succinogenes* [27,28].

Rumen methanogens also participate in symbiotic rela tionships with protozoa that produce large quantities of H_2_ via their hydrogenosomes [24]. In return, the protozoa benefit from H_2_ removal as high H_2_ partial pressure is inhibitory to their metabolism. Meta-analysis of protozoa defaunation studies concluded that elimination of ciliate protozoa reduced CH_4_ production by up to 11% [39]. A similar relationship exists between methanogens and anaerobic rumen fungi which also contain hydrogenosomes [40].

Metatranscriptomic studies [16] showed that, while en zymes mediating fermentative H_2_ production were expressed at similar levels, methanogenesis-related transcripts predominated in high methane yield sheep, while alternative H_2_ uptake pathways were significantly upregulated in low methane yield sheep. These other H_2_ uptake pathways could potentially limit CH_4_ production by redirecting H_2_ uptake away from methanogenesis towards homoacetogenesis (*Blautia*, *Eubacterium*), fumarate and nitrite reduction (*Selenomonas*, *Wolinella*), and sulfate reduction (*Desulfovibrio*). Homoacetogens produce acetate from H_2_ and CO_2_ and are known to occur in the rumen, but their abundance is generally lower than hydrogenotrophic methanogens [41,42]. It is likely that methanogens outcompete homoacetogens at the low H_2_ concentrations in the rumen [43]. Nitrate and sulfate reduction are thermodynamically more favorable than methanogenesis and homoacetogenesis [8], and nitrate and sulfate-reducing bacteria occur naturally in the rumen. Their population densities increase as the concentration of their respective electron acceptors in the ruminant diet increases. However, nitrate and sulfate concentrations in ruminant diets are usually very low so these processes would be substrate limited. Importantly, their end products (nitrite and sulfide) can be toxic at high concentrations [44].

Much less is known about formate concentrations in the rumen or the significance of formate as an electron carrier between species [9]. At relatively high redox potentials (low H_2_ and formate concentrations), formate is thermodynamically and kinetically a more favorable interspecies electron carrier than H_2_. This is of importance mainly for planktonic microbes, where the distances for electron transfer between organisms are greater than between those growing in biofilms and aggregates [45]. Many rumen microbes contain formate dehydrogenase genes, but their expression under different conditions has been much less studied compared to hydrogenase genes.

Fermentation in gut environments/systems can be ex plained thermodynamically as a series of coupled oxidation and reduction reactions, whereby electrons released from fermented substrates are used to reduce electron carriers in microbial cells. During fermentation anaerobic micro-organisms require sinks to dispose of these electrons since the pool of electron carriers is small and finite. If an appropriate electron sink is not available, bacteria cannot regenerate electron carriers at a sufficient rate, metabolic rate is reduced and they are unbale to compete effectively and can get washed out of the rumen. Microbes transfer electrons to other reactions where reducing power is required or to hydrogenases which re-oxidize cofactors reduced during carbohydrate fermentation and dispose of the derived electrons by producing hydrogen (H_2_). Where electrons go can have major implications for the host and in the case of ruminants, the environment as a consequence of GHG emissions.

## WHERE DO ELECTRONS GO DURING RUMEN FERMENTATION?

### Methane

Hydrogen and formate are reduced products of fermentation, and their formation during metabolism and subsequent release into the rumen is a means of electron disposal [10,30]. Methanogenesis is considered to be the main pathway for disposal of H_2_ produced in the rumen [37] with *Methanobrevibacter* spp. the dominant methanogen (Henderson et al [41]). Greening et al [28] showed that electron-bifurcating hydrogenases appear to be key mediators of ruminal H_2_ production. Bacteria belonging to the poorly characterized *Christensenellaceae* R-7 group accounted for a large proportion of these enzymes suggesting that they provide much of the H_2_ used by hydrogenotrophic *Methanobrevibacter* spp suggesting that the interaction between these organisms is an important route of interspecies H_2_ transfer and disposal in the rumen. Based on daily gas production and spot measurements of VFAs, as in the classic balance study of Wolin [46], CH_4_ formation is predicted to account for two-thirds of the electrons generated with approximately 18% of rumen CH_4_ estimated to be produced from formate [9]. However, this is based on a single *in vitro* experiment and requires further measurement and validation as this is likely to be variable. In addition, very little is known about formate concentrations in the rumen and the significance of formate as an intermediate electron acceptor as well as the role of interspecies formate transfer in electron disposal.

### Reduced carbon products

The main products of rumen bacterial carbohydrate fermentation are the major VFAs, acetate, propionate and butyrate. Acetate is more oxidized than the feed being digested, and its formation is associated with electron production. In contrast, propionate and butyrate are significant electron sinks in the rumen ecosystem. In Wolin’s classic rumen fermentation balance experiment [46], these two VFAs account for about one-third of the electrons generated during rumen microbial metabolism (19% and 14%, respectively). This electron disposal route is the most beneficial to the animal as virtually all of the propionate and butyrate is used to support metabolism in the host. This concept is summarized in Figure 3. Other products of rumen microbial fermentation such as succinate, lactate, valerate, caproate, and ethanol are considered minor components because they do not accumulate in significant quantities owing to their rapid turnover and conversion to the three principal VFAs.

### Alternative electron acceptors

Three main pathways for H_2_ disposal in gut systems have been proposed i.e. methanogenesis, sulfate reduction, and reductive acetogenesis [47,48]. In humans, the dominance of these pathways appears to vary among individuals and is influenced by age, diet and many other factors. Furthermore, the quantitative contributions of these three electron disposal routes and the concentrations of these respective electron acceptors H_2_, SO_4_, and CO_2_ + H_2_ have not been measured. Rumen metatranscriptomic and metagenomic studies have shown that in addition to methanogenesis alternative H_2_ uptake pathways, including fumarate, nitrate and sulfate reduction, as well as acetogenesis have been demonstrated [28,38]. Although nitrate and sulfate reduction are thermodynamically more favorable than methanogenesis [8,10], in ruminant diets the concentrations of such electron acceptors are usually very low and are not significant electron sinks unless they are added as dietary supplements [44]. Fumarate and malate can also function as external electron acceptors and are reduced to succinate followed by decarboxylation to propionate. While the rumen environment is considered to be strictly anaerobic, invariably small amounts of oxygen can enter the rumen during ingestion of feed and water, through saliva secretion and by diffusion from blood and rumen epithelial tissues [49]. However, the presence of oxygen is thought to be confined to the superficial layers of the rumen wall and is likely rapidly used by facultative anaerobes and is not a factor in the bulk fermentation.

### Other electron sinks

Other electron sinks include the synthesis of microbial biomass and biohydrogenation whereby unsaturated fatty acids are converted into saturated fatty acids, but these are predicted to be minor sinks relative to CH_4_ and VFAs.

## REDIRECTING HYDROGEN METABOLISM AND RUMINANT METHANE MITIGATION

Currently, there has been increasing urgency in developing approaches that can practically mitigate CH_4_ from ruminant animals [50]. Globally, CH_4_ from enteric fermentation in ruminant livestock is a major source of agricultural GHGs [51, 52]. Ideally, any developed mitigation approach should induce a co-benefit for the animal, for example enhanced production or health. Co-benefits can help drive practical adoption of the technology on farm. While research into reducing ruminant CH_4_ emissions has been in progress for many years [53,54] and promising mitigation approaches are being developed [55], emergence of co-benefits from these approaches is not being observed consistently. This is contrary to the frequently stated hypothesis that ruminal CH_4_ production represents a loss of energy, from 2% to 12% of gross energy intake [56], which could in principle otherwise be available for animal growth or milk production. Historically, it has also been hypothesized that H_2_ accumulation resulting from the inhibition of methanogenesis will impair fiber digestion and fermentation [37]. It is becoming clear that a lack of understanding of H_2_ and formate metabolism and how it can be manipulated is a barrier that needs to be overcome in order to support the development of CH_4_ mitigation approaches with co-benefits. Emerging CH_4_ mitigation strategies in ruminants are now available and can provide model systems to help advance our knowledge in this area. Four promising areas [10,30] are briefly discussed below since there are many other recent reviews that comprehensively describe these nutritional and other methane mitigation strategies. In addition, a summary of methane mitigation strategies for dairy cows is provided in Table 2.

### Animal selection and breeding

Animals vary in their methane production and breeding low methane emitting animals is one mitigation approach. Significant progress has been made with sheep where studies have found animals that vary naturally in the amount of CH_4_ they produce. The heritability of this trait has enabled the breeding of low-CH_4_ emitting sheep [57,58], and CH_4_ emissions from selected, divergent lines differ on average by 10% to 12%. Physiological characteristics such as a reduction in the rumen retention time of feed particles [59] and reduced rumen volume [60] are factors likely to contribute to the low CH_4_ emissions. There are also differences observed in their rumen microbial communities with a *Sharpea*-enriched microbiome characterized by lactate production and utilization [61] and expression of microbial genes involved in the production of CH_4_ are reduced in low-CH_4_ sheep [18]. Kamke et al [62] proposed that the rumen microbiome in low-CH_4_ animals supported heterofermentative growth leading to lactate production, with the lactate subsequently metabolized mainly to butyrate. Greening et al [28] offered an alternative interpretation with H_2_ uptake through non-methanogenic pathways accounting for the differences observed.

### Competing terminal electron acceptors or alternative H_2_ users

Several alternative electron acceptors have been added to ruminant diets in attempts to alter the rumen fermentation and reduce CH_4_ production. Nitrate is the most studied compound [54] and is reduced via nitrite to ammonia, reducing the availability of H_2_ for CH_4_ synthesis. Sulfate reduction will also compete for electrons and H_2_ and may lower CH_4_ production [44]. Stimulating the activity of acetogens through the inhibition of methanogens has been proposed as a strategy for ruminant CH_4_ mitigation [63], but it is unclear whether existing rumen homoacetogens could fulfil the H_2_ disposal role or whether dosed homoacetogens would need to be inoculated into the rumen [64]. Studies to date where methanogenesis has been inhibited with an effective methane inhibitor, such as 3-nitrooxypropanol, have not demonstrated increased homoacetogenesis.

In the gut of macropod marsupials, which consume a diet similar to ruminants, CO_2_ is mainly reduced to acetate rather than to CH_4_ as a means of electron disposal [42]. The reason for the preference of this alternative pathway in macropods is unknown, but it has been argued that their tubiform forestomach lacks the mechanisms to remove gaseous products of fermentation (e.g., eructation in the rumen and flatus in the lower bowel) and their immune secretions suppress the microbes responsible for releasing H_2_ or CH_4_ to prevent gas pressure build-up that would threaten gut integrity [65].

### Methanogen inhibiting technologies

Several different approaches have been used to specifically target methanogens in the rumen. These include feed additives such as 3-nitrooxypropanol [13], halogenated compounds [14], and certain seaweeds [15] as well as work to develop anti-methanogen vaccines [66]. Studies to date with the available technologies suggest that when CH_4_ production is inhibited, we do not observe a sufficient increase in rumen H_2_ emissions to account for the reducing equivalents that are not captured in CH_4_. It is assumed that the electrons are being diverted to other fermentation products, such as acetate, propionate, butyrate, and microbial biomass, but the balance of this redirection of electrons is not well understood. The use of methanogen inhibitors in combination with microbes that could potentially redirect H_2_ to other products has yet to be explored.

An alternative approach is to target the microbes that produce the substrates for methanogenesis. Although recent work has begun to identify the bacteria most likely to produce H_2_ [28] and methyl compounds [67] used as substrates for methanogenesis in the rumen, significant knowledge gaps remain. At this point it is unknown if a reduction in the production of substrates for methanogenesis would decrease overall fermentation.

### Diet

Although methane emissions arising from an individual animal are primarily driven by the quantity of feed eaten [68], the chemical composition of feeds can also influence emissions. Consequently, the nature of the feed consumed may select for microbial populations with different fermentation pathways that yield less H_2_ and therefore less CH_4_. For example, concentrate-based diets are associated with lower CH_4_ yield (g/kg dry matter intake (DMI); [56]) because fermentation of starch in concentrate results in more propionate and butyrate being produced and less CH_4_. Fermentation products [69] and microbial composition [70] may be influenced by the oxidation state of the carbon substrates especially those with higher levels of the more reduced sugar alcohols or more oxidised sugar acids. Brassica forages have also been shown to result in lower CH_4_ yields in lambs than perennial ryegrass [71]. The reason for this is not understood but an altered rumen microbiota or the presence of bioactive glucosinolates in brassicas have been suggested as possible causes [72]. Generally, however, it takes large changes in diet to bring about significant changes in enteric methane emissions in ruminants.

## CONCLUSION

In the rumen, H_2_ and formate have emerged as key metabolites involved in cross-feeding between members of the microbiota with important roles in shaping the syntrophic networks that operate in this ecosystem. The role of H_2_ and formate production as electron sinks for individual microbes and their transfer of electrons to homoacetogenic bacteria and methanogenic archaea are key functions to ensure ongoing polysaccharide degradation and energy generation for ruminants. Although the anaerobic bacteria and archaea are broadly similar in each environment, H_2_ produced in the rumen is consumed predominantly by incorporation into CH_4_, whereas in the human colon significant H_2_ emissions escape the system. Determining what controls these differences will be important in understanding the impact of H_2_ and formate turnover on rumen metabolism and fermentation and in reducing the environmental impact of ruminant CH_4_ emissions. Currently, the availability of emerging CH_4_ mitigation approaches for ruminant animals makes the rumen an ideal gut system to study the production and utilization of hydrogen and formate in gut systems and generate knowledge applicable to both systems.

Our present knowledge of rumen H _2_ and formate economy is incomplete and an improved understanding of the active groups of microbes involved in H_2_, and formate metabolism is required. Cultures and genome sequences of model hydrogenotrophs, such as *Methanobrevibacter*, and *Blautia*, are available and have been used to demonstrate interspecies H_2_ and formate transfer in co-culture. However, our knowledge of which organisms produce the bulk of the H_2_ and formate in the rumen remains limited. The metagenome-and metatranscriptome-based studies have highlighted the diversity of gut microbes encoding the signature genes for hydrogenotrophy and have emphasized the need for more exact information about the function of these genes in the gut environment. There also remains a need to bring a greater proportion of representatives of the currently uncultured microorganisms into cultivation together with additional host-associated homoacetogen and methanogen strains. This should be accompanied by studies of their physiology, metabolism, and interactions with other gut anaerobes to provide a body of knowledge beyond what can be inferred from genome and metagenome sequence data. With the increased availability of such pure cultures and their corresponding genome sequences it will prove possible to construct metabolically interacting microbial consortia so that the contributions of different microbes to overall community function can be ascertained.

## Figures and Tables

**Table 1:** Stoichiometric amounts of methane produced per mole hexose fermented in ruminal mixed acid fermentations [7]

Molar ratio (acetate:propionate:butyrate)	Mole CH_4_ produced per mole hexose fermented
65:20:15	0.61
60:25:15	0.54
55:30:15	0.48
70:20:10	0.64
65:25:10	0.57
60:30:10	0.50

**Table 2:** Methods of reducing methane emissions from dairy cows and expected timeline for implementation [73,74]

Timeline for development	Mitigation practice for the dairy industry	Expected reduction in methane (%)
Immediate	Feeding oils and oilseeds	5 – 20
	Higher grain diets	5 – 10
	Using legumes rather than grasses	5 – 15
	Using corn silage or small grain silage rather than grass silage or grass hay	5 – 10
	Ionophores	5 – 10
	Herd management to reduce animal numbers	5 – 20
	Best management practices that increase milk production per cow	5 – 20
3 to 5 years	Rumen modifiers (yeast, enzymes, directly fed microbials)	5 – 15
	Plant extracts (tannins, saponins, oils)	5 – 20
	Animal selection for increased feed conversion efficiency	10 – 20
	3-Nitrooxy propanol (3-NOP) - methanogenesis inhibitor	30 – 40
	Marine algae (bromoforms)	30
>10 years	Vaccines	10 – 20
	Strategies that alter rumen microbial populations	30 – 60
